# Double knockout CRISPR screen for cancer resistance to T cell cytotoxicity

**DOI:** 10.1186/s13045-022-01389-y

**Published:** 2022-12-01

**Authors:** Jonathan J. Park, Adan Codina, Lupeng Ye, Stanley Lam, Jianjian Guo, Paul Clark, Xiaoyu Zhou, Lei Peng, Sidi Chen

**Affiliations:** 1 Department of Genetics, Yale University School of Medicine, New Haven, CT USA; 2 System Biology Institute, Yale University, West Haven, CI USA; 3 Center for Cancer Systems Biology, Yale University, West Haven, CI USA; 4 Combined Program in the Biological and Biomedical Sciences, Yale University, New Haven, CT USA; 5 Molecular Cell Biology, Genetics, and Development Program, Yale University, New Haven, CT USA; 6 MD-PhD Program, Yale University, New Haven, CT USA; 7 The College, Yale University, New Haven, CT USA; 8 Department of Neurosurgery, Yale University School of Medicine, New Haven, CT USA; 9 Immunobiology Program, Yale University, New Haven, CT USA; 10 Comprehensive Cancer Center, Yale University School of Medicine, New Haven, CT USA; 11 Stem Cell Center, Yale University School of Medicine, New Haven, CT USA; 12 Liver Center, Yale University School of Medicine, New Haven, CT USA; 13 Center for Biomedical Data Science, Yale University School of Medicine, New Haven, CT USA; 14 Center for RNA Science and Medicine, Yale University School of Medicine, New Haven, CT USA

**Keywords:** CRISPR screen, Immunotherapy, Cancer immunology, Genetic interaction, Double knockout, Systems biology

## Abstract

Immunotherapy has transformed cancer treatments; however, a large fraction of patients encounter resistance. Such resistance is mediated by complex factors, often involving interactions between multiple genes. Thus, it is crucially important to identify genetic interactions between genes that are significantly mutated in cancer patients and those involved in immune responses, ideally the ones that currently have chemical compounds for direct targeting. To systematically interrogate such genetic interactions that mediate cancer cells’ response to T cell killing, we designed an asymmetric dual perturbation library targeting the matched combinations between significantly mutated tumor suppressors and immune resistance genes. We performed a combinatorial double knockout screen on 1159 gene pairs and identified those where joint loss-of-function renders altered cellular response to T cell cytotoxicity. We also performed comparative transcriptomics-based analyses on tumor and normal samples from TCGA and GTEx cohorts, mutational profiling analyses, and survival analyses to further characterize the significance of identified hits in clinical patients. Interactions between significantly mutated tumor suppressors and potentially druggable immune resistance genes may offer insights on potential new concepts of how to target clinically relevant cancer mutations with currently available agents. This study also provides a technology platform and an asymmetric double knockout library for interrogating genetic interactions between cancer mutations and immune resistance pathways under various settings.

**Supplementary Information:**

The online version contains supplementary material available at 10.1186/s13045-022-01389-y.

## To the editor,

**Fig. 1:**
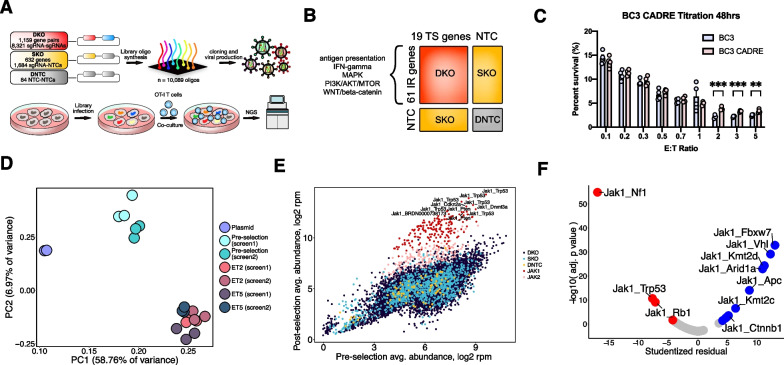
Asymmetric double knockout CRISPR screen of gene pairs that affect cancer cell response to T cell killing. **A** Schematic overview of CADRE screen. **B** Schematic of CADRE library design, 61 genes with immunotherapy resistance were crossed in combinatorial fashion with 19 significantly mutated tumor suppressors to create a DKO pool. SKOs and NTCs serve for comparison and as controls. **C** Titration of BC3 cells, and BC3 CADRE cells co-cultured with E:T ratios ranging from 0.1 to 5. High E:T ratios demonstrated significant phenotypic differences and were therefor selected for screening (q values of 1.76e-3 and 1.19e-2 by multiple T test, 1% FDR for E:T ratios 2 and 5, respectively). *, adjusted *p*-value < 0.05. **, adjusted *p*-value < 0.01. ***, adjusted *p*-value < 0.001. **D** Principal component analysis (PCA) of the sgRNA pair read count distributions across screens, E:T ratios, technical replicates, and pre-T cell treatment controls. **E** Scatterplots comparing guide representation of the CADRE library in post co-culture samples averaged across all replicates, E:T ratios, and screens compared to pre-selection infected cell controls. *Jak1*- and *Jak2-*associated sgRNA pairs (either DKO or SKO) are marked in red. **F** Scatterplot comparing Bonferroni-adjusted *p* value determined by outlier test compared to Studentized residuals from linear regression analysis in Additional file 1: Fig. S3F

Despite impressive durable responses elicited by cancer immunotherapy, the majority of patients do not see long-term benefit with treatment [1, 2]. However, the molecular mechanisms that determine therapeutic resistance remain poorly understood, particularly genetic interactions. To systematically interrogate such genetic interactions that mediate immune resistance, we designed a Combinatorial Antineoplastic Drug Resistance Experiment (CADRE) screening strategy with an asymmetric library design (Fig. 1A, B). The CADRE library was synthesized via oligonucleotide array and cloned into lentiviral vectors (Additional file 1: Fig. S1A), and the representations of double knockouts (DKOs), single knockouts (SKOs), and double non-targeting controls (DNTCs) in the library were verified by next-generation sequencing (NGS) (Additional file 1: Fig. S1C, D). We transduced B16F10;OVA;Cas9 cells at a low multiplicity of infection (MOI) (MOI < 0.2) at a coverage of approximately 500X. We NGS verified that the transduced pre-selection cell pool retained the vast majority of the CADRE library (Additional file 1: Fig. S1E).

We then performed co-culture assays on library and non-library infected B16F10;OVA;Cas9 clone #3 cells (**BC3** cells) with OT-I CD8 + T cells. Both library and non-library transduced cells showed comparable survival across E:T ratios, with the exception of at the high E:T ratio conditions (E:T ratios > 1) where the mutant pool demonstrated a significant increase in resistance (Fig. 1C). We then performed the co-culture screen with BC3-CADRE cells and OT-I CD8 + T-cells, followed by library NGS readout (Additional file 1: Fig. S2A). Clustering analysis showed distinct clusters between plasmid, cell populations before co-culture, and cell populations post co-culture (Additional file 1: Fig. S2B), suggesting a high-quality screen and NGS readout between the cell pool conditions and E:T ratios 2–5 (Fig. 1D). There are strong shifts between pre-selection and post-selection co-cultures (Additional file 1: Fig. S2C), indicative of strong selection seen at sgRNA library levels.

**Fig. 2:**
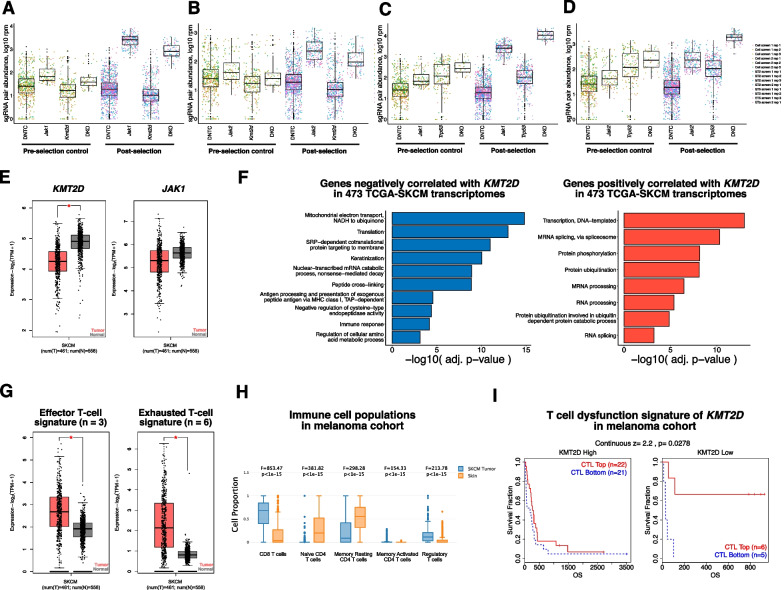
Genetic analysis of gene pairs that affect cancer cell response to T cell killing. **A**–**D** Tukey box plots (IQR boxes with 1.5 × IQR whiskers) overlaid on dot plots of sgRNA pair abundances for each DKO, SKO, and DNTCs for pre-T cell treatment controls (also labeled as “cell”) and post-selection co-culture samples with reads pooled from samples across screens, E:T ratios, and technical replicates. Count distributions are shown for gene pairs *Jak1_Kmt2d*, *Jak2_Kmt2d*, *Jak1_Trp53*, and *Jak2_Trp53*. **E** Boxplots of *KMT2D* and *JAK1* expression in RNA-seq samples from the TCGA SKCM melanoma cohort and paired normal samples (461 tumor samples, 558 normal samples). *, q-value < 0.01 and |log2FC|> 0.5. **F** Bar plots of top enriched pathways identified by DAVID biological processes analyses of genes negatively (left) and positively (right) correlated with *KMT2D* expression in 473 melanoma RNA-seq samples from TCGA. **G** Boxplots of effector T-cell gene signatures (*CX3CR1, FGFBP2, FCGR3A*) and exhausted T-cell gene signatures (*HAVCR2, TIGIT, LAG3, PDCD1, CXCL13, LAYN*) in RNA-seq samples from the TCGA SKCM melanoma cohort and paired normal samples (461 tumor samples, 558 normal samples). *, q-value < 0.01 and |log2FC|> 0.5. **H** Boxplots of normalized cell type proportions from CIBERSORT deconvolution analyses of TCGA-SKCM and GTEx normal skin RNA-seq samples for T cells. Statistics shown on plots. **I** Kaplan–Meier curves showing the survival of melanoma patients from the GSE22153 cohort based on the expression status of *KMT2D* and linked to estimated cytotoxic T lymphocyte (CTL) levels. Analyses performed with the Tumor Immune Dysfunction and Exclusion (TIDE) algorithm. Statistics shown on plot

At a false-discovery rate (FDR) of 1.19% we identified 222 enriched sgRNA pairs of which 194 (87.4%) are associated with *Janus kinase 1 (Jak1)* or *Janus kinase 2 (Jak2)*, including DKO and SKO constructs. Bulk analysis revealed that *Jak*-associated sgRNAs dominated the enrichment in the screen post-selection (Fig. 1E; Additional file 1: Fig. S3A–D). We found that *Jak1/2*-associated gene pairs were the most statistically significantly different from their constitutive SKOs (Fig. 1E, F; Additional file 1: Fig. S3E, F), suggestive of potential gene interactions. We observe that gene pairs *Jak1_Trp53, Jak1_Nf1, and Jak1_Rb1* have higher observed enrichment for double knockout than expected (adjusted *p*-value < 0.001) suggesting potential additive gene interaction (Fig. 1F), while gene pairs *Jak1_Apc, Jak1_Vhl, Jak1_Kmt2c, Jak1_Kmt2d, Jak1_Arid1a, Jak1_Fbxw7, Jak1_Ctnnb1* have lower observed enrichment for double knockout than expected, suggesting potential subtractive gene interaction (Fig. 1F). Boxplots of normalized read counts for *Jak1_Kmt2d, Jak2_Kmt2d, Jak1_Trp53* and *Jak2_Trp53* (Fig. 2A–D) also suggest potential subtractive and additive phenotypic interactions to *Jak1/2* perturbation for *Kmt2d* and *Trp53*, respectively. However, it should be noted that although significant, the putative gene interaction signals appear to be modest in part due to the strong resistance phenotype of single knockout of *JAK1/2*.

We looked at the global gene expression profiles of *KMT2D*, *JAK1*, *TP53*, and *IFNGR1* across all tumor samples and paired normal tissues (Additional file 1: Fig. S4A–D) and more specifically for *KMT2D* and *JAK1* in the SKCM cohort (Fig. 2E–G) and identified tumor-type specific expression patterns. We found that the effector and exhaustion T cell signatures were upregulated in the tumor samples in melanoma patients (Fig. 2G). Cell proportion deconvolution analyses revealed increased estimated proportions of CD8 T cells, memory-activated CD4 T cells, and Tregs in the tumor samples, with a decrease of naïve and memory resting CD4 T cells (Fig. 2H).

Genes negatively correlated with *KMT2D* were further analyzed using DAVID gene ontology functional annotation (Fig. 2F). We found positive and significant correlations for both *JAK1* and IFN-gamma signaling gene signatures across both the SKCM cohort and across 33 different cancer types from TCGA (Additional file 1: Fig. S5A). *KMT2D* and *JAK1* are both frequently mutated in melanoma patients (Additional file 1: Fig. S5B). Mutual exclusivity and co-occurrence analyses for all pairwise combinations of *KMT2D*, *JAK1*, *JAK2*, *IFNGR1*, and *TP53* suggest that all mutation combinations except *JAK2-IFNGR1* co-occur at a significant rate (Additional file 1: Fig. S5C).

We also performed survival analyses on patient cohorts with the public database TCGA (The Cancer Genome Atlas) (Additional file 1: Figs. S4E, F, S5D, E). Survival maps (Additional file 1: Fig. S4G, H) revealed cancer-type specific effects of *KMT2D*, *JAK1*, *JAK2*, *IFNGR1*, or *TP53* expression levels on patient survival. The *KMT2D*-low patient group demonstrated increased CTL-associated overall survival benefit, whereas high levels of *KMT2D* abolished the overall survival benefit of CTL-high patients (Fig. 2I).

Altogether, we demonstrate how dual loss-of-function CRISPR screens with asymmetric library designs can resolve complex phenotypes such as resistance to T cell killing.

## Supplementary Information

**Additional file 1. **Supplemental figures.

**Additional file 2. **Supplemental tables and datasets.

**Additional file 3. **Additional supplemental materials including methods, figure legends, list of tables, and references.

## Data Availability

All data generated or analyzed during this study are included in this article and its Additional files. Specifically, source data and statistics for non-high-throughput experiments are provided in Additional file 2: Tables and Datasets. Processed data for high-throughput sequencing experiments are provided as processed quantifications in Additional file 2: Tables and Datasets. Raw sequencing data is available via SRA/BioProject under accession number PRJNA661532. Original cell lines are available at commercial sources listed in Additional files. Genetically modified cell lines are available via Chen lab. Most data, reagents, methods, computational codes and materials that support the findings of this research are available from the corresponding author upon reasonable request.

## Abbreviations

BC3 cells: B16F10;OVA;Cas9 clone #3 cells
FLuc: Firefly luciferase
CADRE: Combinatorial antineoplastic drug resistance experiment
SMG: Significantly mutated gene
LOF: Loss of function
GEMM: Genetically engineered mouse model
CTL: Cytotoxic T lymphocyte
ICB: Immune checkpoint blockade
TIDE: Tumor immune dysfunction and exclusion
DAVID: Database for annotation, visualization and integrated discovery
TCGA: The cancer genome Atlas
SKCM: Skin cutaneous melanoma
GTEx: Genotype-tissue expression
OVA: Ovalbumin
DKO: Double knockout
SKO: Single knockout
NTC: Non-targeting control
DNTC: Double non-targeting control
