# Supplementary material for: Double knockout CRISPR screen for cancer resistance to T cell cytotoxicity
Source: J Hematol Oncol. 2022 Dec 1;15:172. doi: 10.1186/s13045-022-01389-y (PMC9716677; doi:10.1186/s13045-022-01389-y)

Figure S1

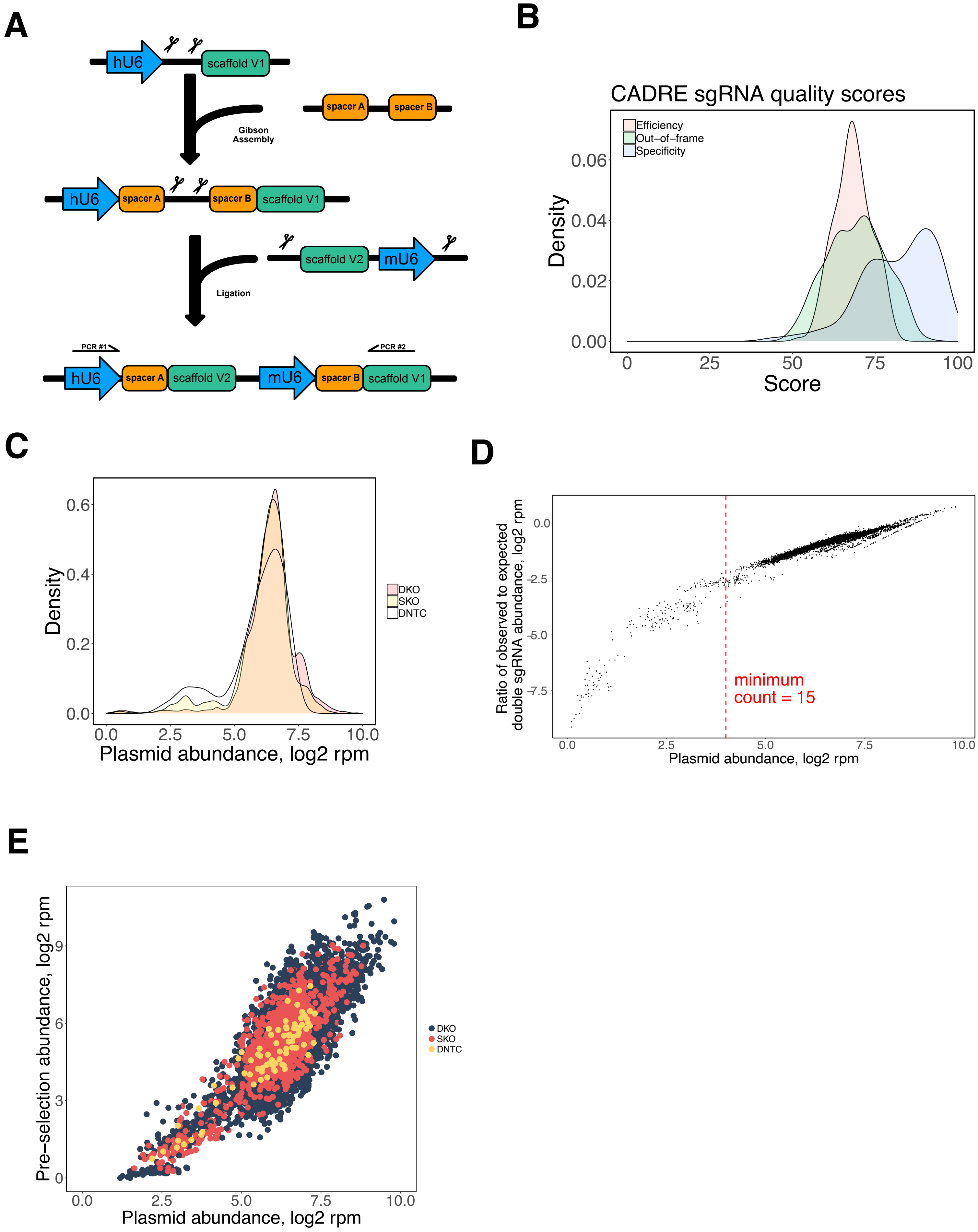

A
hU6
scaffold V1
spacer A
spacer B
Gibson Assembly
Ligation
scaffold V2
mU6
PCR #1
PCR #2
B
CADRE sgRNA quality scores
Efficiency
Out-of-frame
Specificity
Density
Score
C
Density
DKO
SKO
DNTC
Plasmid abundance, log2 rpm
D
Ratio of observed to expected double sgRNA abundance, log2 rpm
minimum count = 15
Plasmid abundance, log2 rpm
E
Pre-selection abundance, log2 rpm
DKO
SKO
DNTC
Plasmid abundance, log2 rpm

Figure S2

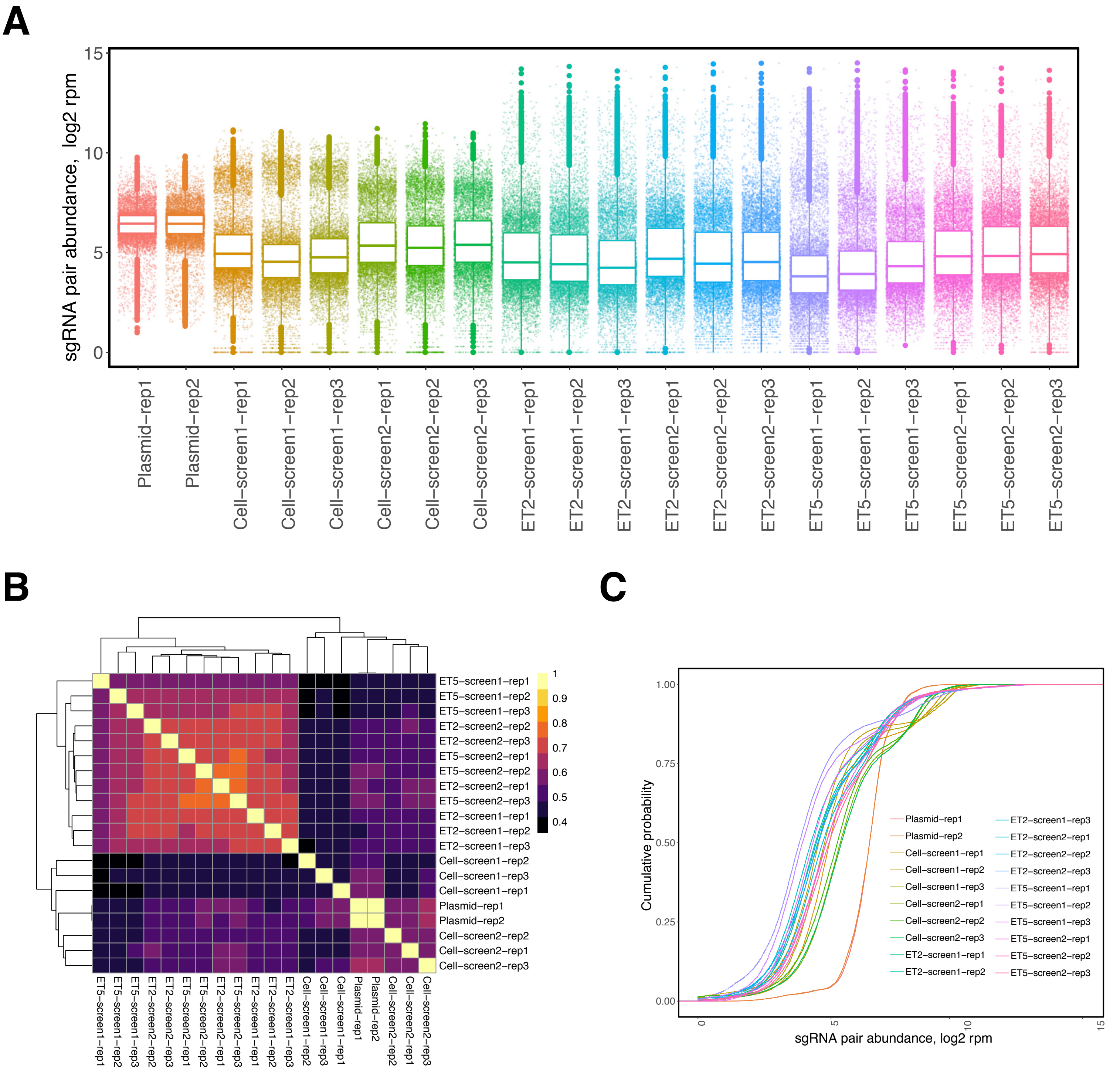

A
sgRNA pair abundance, log2 rpm
0
5
10
15
Plasmid−rep1
Plasmid−rep2
Cell−screen1−rep1
Cell−screen1−rep2
Cell−screen1−rep3
Cell−screen2−rep1
Cell−screen2−rep2
Cell−screen2−rep3
ET2−screen1−rep1
ET2−screen1−rep2
ET2−screen1−rep3
ET2−screen2−rep1
ET2−screen2−rep2
ET2−screen2−rep3
ET5−screen1−rep1
ET5−screen1−rep2
ET5−screen1−rep3
ET5−screen2−rep1
ET5−screen2−rep2
ET5−screen2−rep3
B
ET5−screen1−rep1
ET5−screen1−rep2
ET5−screen1−rep3
ET2−screen2−rep2
ET2−screen2−rep3
ET5−screen2−rep1
ET5−screen2−rep2
ET2−screen2−rep1
ET5−screen2−rep3
ET2−screen1−rep1
ET2−screen1−rep2
ET2−screen1−rep3
Cell−screen1−rep2
Cell−screen1−rep3
Cell−screen1−rep1
Plasmid−rep1
Plasmid−rep2
Cell−screen2−rep2
Cell−screen2−rep1
Cell−screen2−rep3
1
0.9
0.8
0.7
0.6
0.5
0.4
C
Cumulative probability
0.00
0.25
0.50
0.75
1.00
sgRNA pair abundance, log2 rpm
0
5
10
15
Plasmid−rep1
Plasmid−rep2
Cell−screen1−rep1
Cell−screen1−rep2
Cell−screen1−rep3
Cell−screen2−rep1
Cell−screen2−rep2
Cell−screen2−rep3
ET2−screen1−rep1
ET2−screen1−rep2
ET2−screen1−rep3
ET2−screen2−rep1
ET2−screen2−rep2
ET2−screen2−rep3
ET5−screen1−rep1
ET5−screen1−rep2
ET5−screen1−rep3
ET5−screen2−rep1
ET5−screen2−rep2
ET5−screen2−rep3

Figure S3

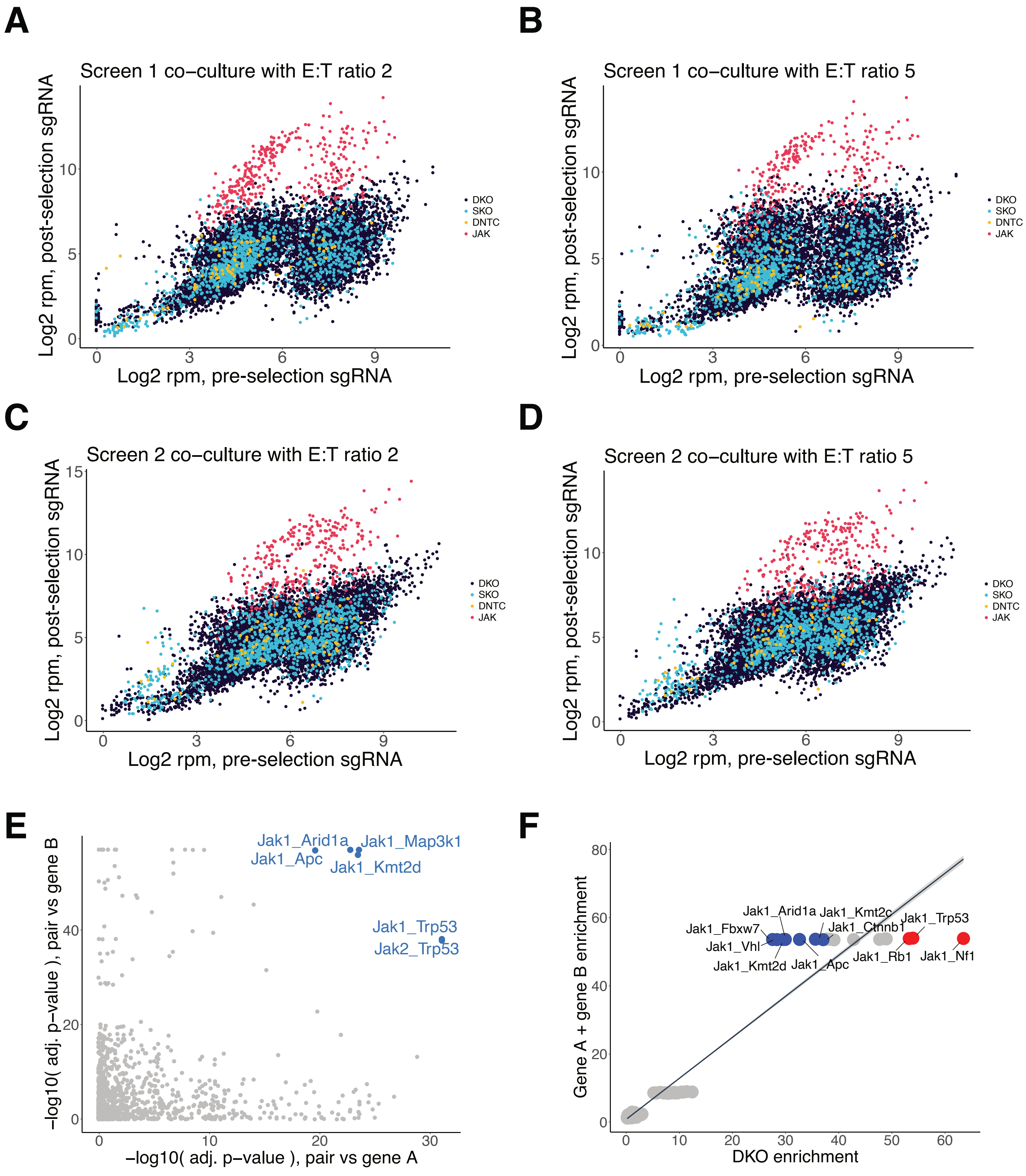

A
Screen 1 co−culture with E:T ratio 2
Log2 rpm, post-selection sgRNA
Log2 rpm, pre-selection sgRNA
DKO
SKO
DNTC
JAK
B
Screen 1 co−culture with E:T ratio 5
Log2 rpm, post-selection sgRNA
Log2 rpm, pre-selection sgRNA
DKO
SKO
DNTC
JAK
C
Screen 2 co−culture with E:T ratio 2
Log2 rpm, post-selection sgRNA
Log2 rpm, pre-selection sgRNA
DKO
SKO
DNTC
JAK
D
Screen 2 co−culture with E:T ratio 5
Log2 rpm, post-selection sgRNA
Log2 rpm, pre-selection sgRNA
DKO
SKO
DNTC
JAK
E
Jak1_Arid1a
Jak1_Map3k1
Jak1_Apc
Jak1_Kmt2d
Jak1_Trp53
Jak2_Trp53
−log10(adj. p−value), pair vs gene B
−log10(adj. p−value), pair vs gene A
F
Jak1_Arid1a
Jak1_Kmt2c
Jak1_Trp53
Jak1_Fbxw7
Jak1_Ctnnb1
Jak1_Vhl
Jak1_Kmt2d
Jak1_Apc
Jak1_Rb1
Jak1_Nf1
Gene A + gene B enrichment
DKO enrichment

Figure S4

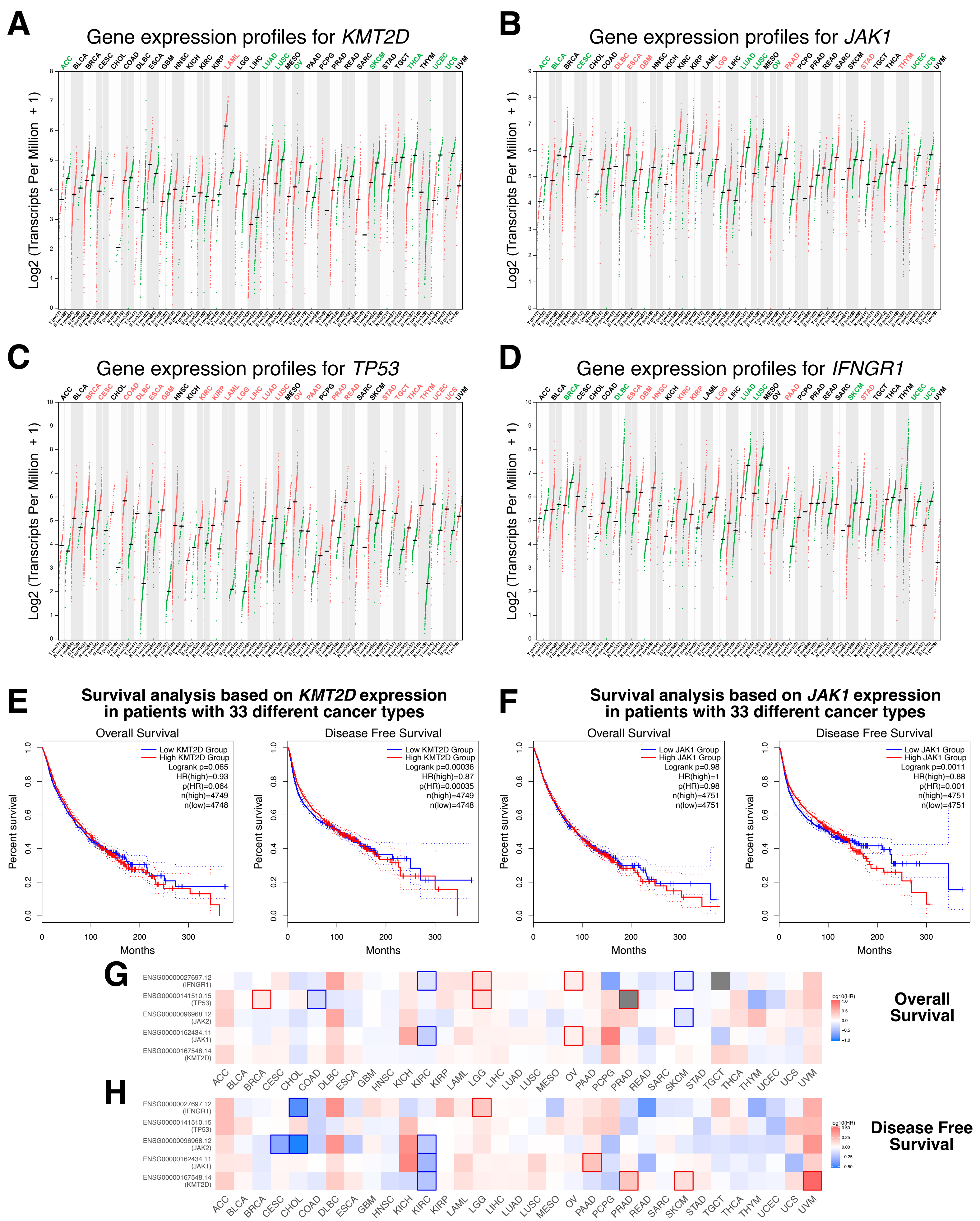
A
Gene expression profiles for KMT2D
B
Gene expression profiles for JAK1
C
Gene expression profiles for TP53
D
Gene expression profiles for IFNGR1
Log2 (Transcripts Per Million + 1)
ACC BLCA BRCA CESC CHOL COAD DLBC ESCA GBM HNSC KICH KIRC KIRP LAML LGG LIHC LUAD LUSC MESO OV PAAD PCPG PRAD READ SARC SKCM STAD TGCT THCA THYM UCEC UCS UVM
E
Survival analysis based on KMT2D expression in patients with 33 different cancer types
Overall Survival
Low KMT2D Group
High KMT2D Group
Logrank p=0.065
HR(high)=0.93
p(HR)=0.064
n(high)=4749
n(low)=4748
Disease Free Survival
Low KMT2D Group
High KMT2D Group
Logrank p=0.00036
HR(high)=0.87
p(HR)=0.00035
n(high)=4749
n(low)=4748
F
Survival analysis based on JAK1 expression in patients with 33 different cancer types
Overall Survival
Low JAK1 Group
High JAK1 Group
Logrank p=0.98
HR(high)=1
p(HR)=0.98
n(high)=4751
n(low)=4751
Disease Free Survival
Low JAK1 Group
High JAK1 Group
Logrank p=0.0011
HR(high)=0.88
p(HR)=0.001
n(high)=4751
n(low)=4751
Percent survival
Months
G
ENSG00000027697.12 (IFNGR1)
ENSG00000141510.15 (TP53)
ENSG00000096968.12 (JAK2)
ENSG00000162434.11 (JAK1)
ENSG00000167548.14 (KMT2D)
log10(HR)
Overall Survival
H
Disease Free Survival

Figure S5

A

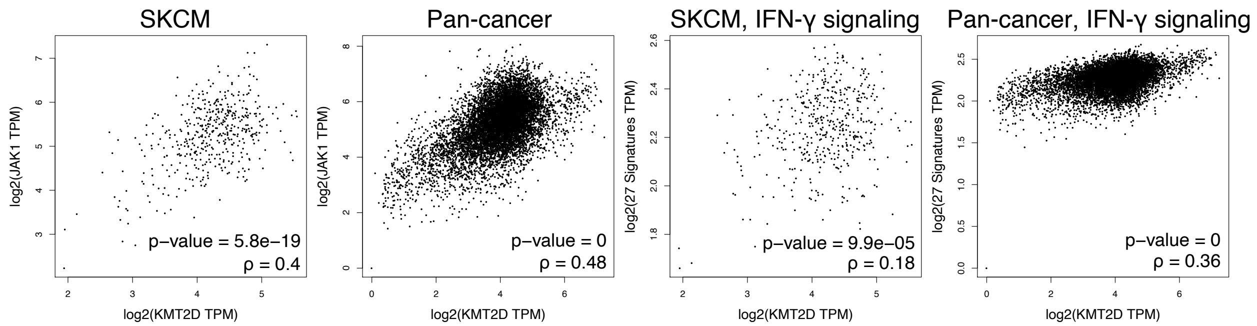

B

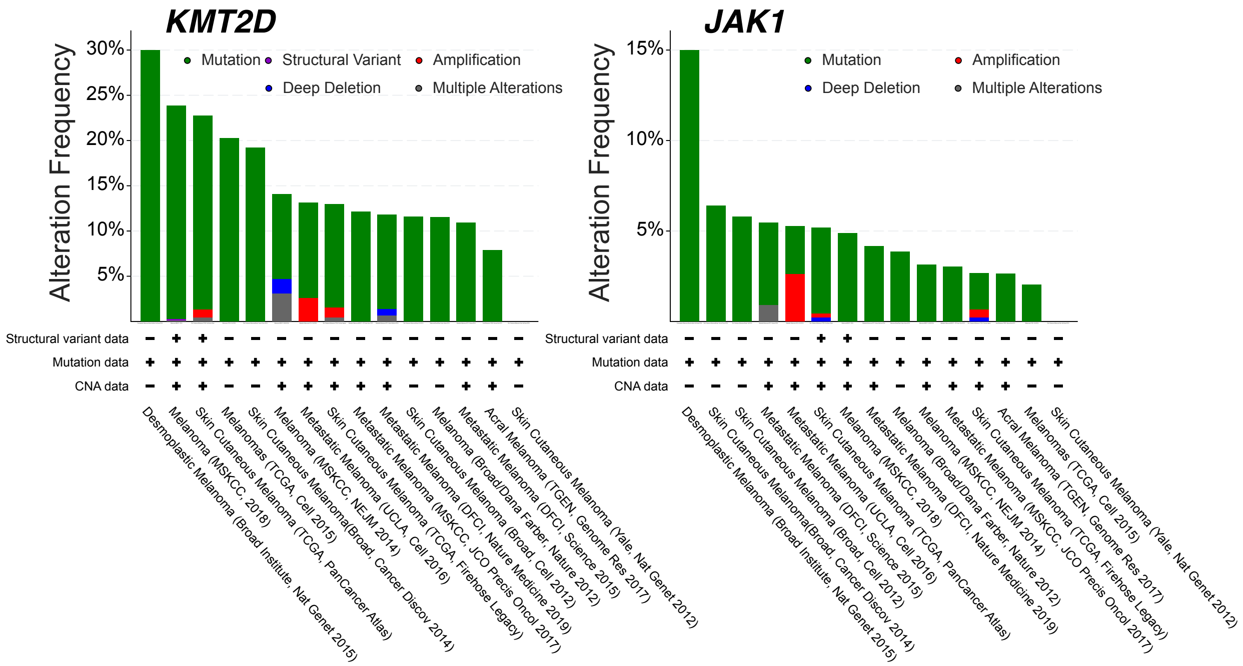

C

A	B	Neither	A Not B	B Not A	Both	Log2 Odds Ratio	p-Value	q-Value	Tendency
KMT2D	TP53	1755	312	326	150	1.372	<0.001	<0.001	Co-occurrence
KMT2D	JAK2	2012	411	69	51	1.855	<0.001	<0.001	Co-occurrence
JAK1	TP53	1999	68	436	40	1.431	<0.001	<0.001	Co-occurrence
JAK2	TP53	1990	77	433	43	1.36	<0.001	<0.001	Co-occurrence
JAK1	JAK2	2330	93	105	15	1.84	<0.001	<0.001	Co-occurrence
KMT2D	JAK1	2008	427	73	35	1.173	<0.001	<0.001	Co-occurrence
KMT2D	IFNGR1	1988	432	35	22	1.532	<0.001	<0.001	Co-occurrence
JAK1	IFNGR1	2322	98	49	8	1.952	0.002	0.003	Co-occurrence
IFNGR1	TP53	1973	38	447	19	1.142	0.006	0.007	Co-occurrence
JAK2	IFNGR1	2310	110	52	5	1.014	0.122	0.122	Co-occurrence

D

Survival analysis based on *KMT2D* expression in TCGA-SKCM patients

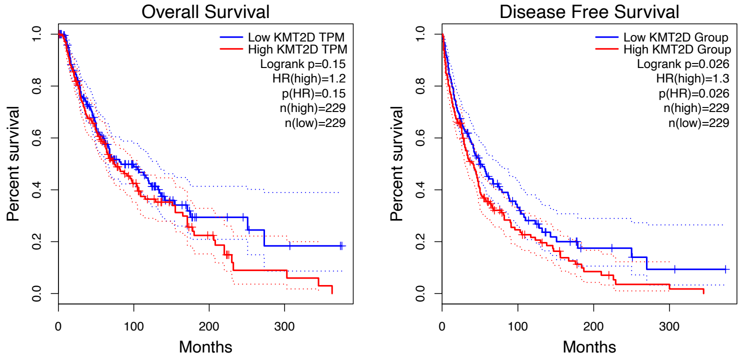

E

Survival analysis based on *JAK1* expression in TCGA-SKCM patients

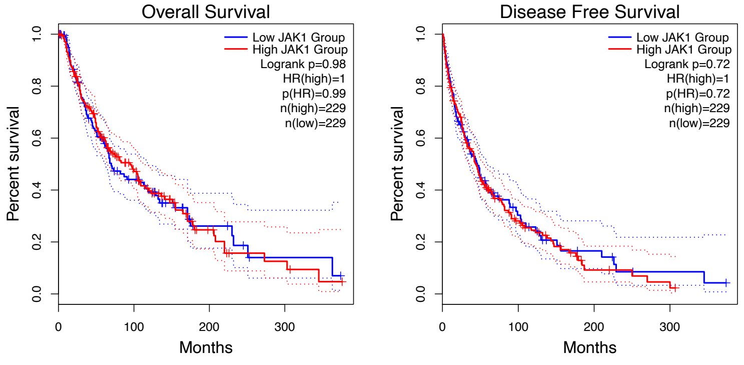

Supplement: Supplementary file 1 — Additional file 1. Supplemental figures. [file 13045_2022_1389_MOESM1_ESM.pdf]
