# Supplementary material for: Double knockout CRISPR screen for cancer resistance to T cell cytotoxicity
Source: J Hematol Oncol. 2022 Dec 1;15:172. doi: 10.1186/s13045-022-01389-y (PMC9716677; doi:10.1186/s13045-022-01389-y)
Supplement: Supplementary file 3 — Additional file 3. Additional supplemental materials including methods, figure legends, list of tables, and references. [file 13045_2022_1389_MOESM3_ESM.docx]

**Supplemental Materials for**

**Double knockout CRISPR screen in cancer resistance to T cell cytotoxicity**

Jonathan J. Park ^1,2,3,4,5,6*^, Adan Codina ^1,2,3,4,5,*^, Lupeng Ye ^1,2,3,*^,

Stanley Lam ^1,2,3,7^, Jianjian Guo ^1,2,3,4,5^, Paul Clark ^1,2,3^, Xiaoyu Zhou ^1,2,3^, Lei Peng ^1,2,3^,

and Sidi Chen ^1,2,3,4,5,6,8,9,10,11,12,13,14,#^

**Methods:**

**Cell lines**

Single cell clones were derived from the murine melanoma cell line (B16F10) transduced with PGK-mCherry-OVA lentivirus to reduce cellular heterogeneity and ensure homogenous expression of antigen. Single cells were sorted and multiple clones were derived. A clonal cell line (clone #3) was used for subsequent experiments. A combination of antibiotic selection and flow cytometry was used to ensure purity. These cells were subsequently transduced with Cas9 lentivirus to create B16F10;OVA;Cas9 clone #3 cells (referred to as BC3 cells). All screens were conducted in this background. BC3 cells were transduced with Firefly Luciferase (FLuc) lentivirus for bioluminescence assays, and all experiments were conducted in the BC3-FLuc background. All cell lines were grown under standard conditions using D10 (DMEM supplemented with 10% FBS, 1% Penicillin-Streptomycin) in an incubator maintained at 37 ℃ with 5% CO_2_.

**Design of CADRE library**

The Combinatorial Antineoplastic Drug Resistance Experiment (CADRE) library follows an asymmetric design, combining the most significantly mutated tumor suppressors in human cancers with immunotherapy resistance associated genes derived from the antigen processing and presentation, IFN-gamma, MAPK, PI3K/AKT/MTOR, and WNT/beta-catenin signaling pathways. To generate the immunotherapy resistance gene set, the union of the following gene lists were used “regulation_of_MAP_kinase_activity” (GO: 0043405), “antigen_processing_and_presentation” (GO: 0019882), “interferon-gamma-mediated_signaling_pathway” (GO: 0060333), “T_cell_costimulation” (GO: 0031295), “HALLMARK_PI3K_AKT_MTOR_SIGNALING” (MSigDB: M5923), “HALLMARK_WNT_BETA_CATENIN_SIGNALING” (MSigDB: M5895), “HALLMARK_INTERFERON_GAMMA_RESPONSE” (MSigDB: M5913), with the following gene lists removed “T_cell_receptor_complex” (GO: 0042101), “B_cell_receptor_signaling_pathway” (GO: 0050853), “Toll-like_receptor_1-Toll-like_receptor_2_protein_complex” (GO: 0035354), “Toll-like_receptor_2-Toll-like_receptor_6_protein_complex” (GO: 0035355). This gene list was then intersected with the a list of drug targets obtained by cross-reference of the UniProtKB/Swiss-Prot manually reviewed, complete human proteome (proteome identifier up000005640) with the DrugBank database of targets for FDA-approved small molecule drugs, FDA-approved biotech drugs, nutraceuticals, and experimental drugs. The tumor suppressor gene list was curated from Kandoth et al., 2013 ^1^. sgRNAs were designed using a custom pipeline integrating sgRNA quality scoring by CRISPOR ^2^. In total, the library consists of 1,159 DKO gene pairs represented by 8,321 DKO sgRNA-sgRNAs; 632 SKO genes represented by 1,684 sgRNA-NTCs; and 84 DNTCs. The library was synthesized as an oligo pool (CustomArray).

**Lentiviral production and transduction**

Prior to transduction, media was removed from 80-90 percent confluent HEK293FT cells and replaced with OptiMEM serum free media to enhance transduction efficiency. Envelope packaging plasmid pMD2.G, packaging plasmid psPAX2, and dual-sgRNA lentiviral plasmids were combined in a ratio of 1:1.5:2 and suspended in OptiMem serum free media. Polyethyleneimine (PEI) was added to plasmid DNA pool (8 μL 1mg/mL PEI per 1μg DNA) and mixed gently before being incubated at room temperature for 15 min. After incubation, plasmid solutions were added dropwise to HEK293FT cells. 6 hours post transduction OptiMEM media was replaced with D10 media. Supernatant was collected from HEK293FT cells after 48 h post transduction and spun down at 1000 x *g*, 4 ℃, for 5 min to remove cellar debris. Viral supernatant was then aliquoted before being frozen at -80 ℃ prior to experimentation. To determine viral titer to be infected cells were counted and seeded in plates at appropriate densities, before being infected with various dilutions of viral supernatant. 24 h post infection titrated puromycin was added to infected cells (10 μg/mL) and cultured for 72 h. Cell survival was then assayed to determine the functional viral titer of the supernatant.

**Double knockout cellular library production**

Each screen was performed with four infection replicates, low multiplicity of infection (MOI), and high screening coverage. Briefly, BC3 cells were seeded at a density of 5e6 cells per plate in 15cm plates were transduced with 1e6 functional viral particles per plate for a calculated MOI of 0.2, and incubated for 24 h prior to replacing media with fresh media containing 4 μg/mL puromycin for selection. A total of 2.5e7 cells (5 plates of 5e6) were seeded and approximately a total of 5e6 cells were infected, conferring ~500x library coverage.

**Naïve OT-I CD8^+^ T cell isolation and culture**

Mouse CD8^+^ T cell isolation and culture methods were based on our previous work ^3,4^. Briefly, mesenteric lymph nodes (mLNs) and spleens were dissected from OT-I mice, then placed into ice-cold PBS supplemented with 2 % FBS. Lymphocytes were released by grinding organs through a 100 μm filter, then re-suspended with 2 % FBS. Red blood cells (RBCs) were lysed with ACK lysis buffer (Lonza). RBC-lysed lymphocyte solution was filtered through 40 μm filters to remove cell debris. Naïve CD8a^+^ T cell purification was performed using Naïve CD8a^+^ T cell Isolation Kits (Miltenyi Biotec) according to the manufacturer’s protocols. Naïve CD8a^+^ T cells were cultured in RPMI-1640 (Gibco) media supplemented with 10 % FBS, 2 mM L-Glutamine, 200 U / mL penicillin–streptomycin (Gibco), and 49 μM β-mercaptoethanol (Sigma), hereafter referred to as cRPMI media. Naïve CD8a^+^ T cells were activated with anti-CD3ε and anti-CD28 antibodies (BioLegend), For *in vitro* experiments, cRPMI media was supplemented with 2 ng / mL IL-2, 1 μg / mL anti-CD28, and 12 ng / mL Il-12p70 cytokines or antibodies. All cytokines and antibodies were purchased from BioLegend. For antigen stimulation co-culture experiments OT-1 cells were collected as described above but activated by culturing harvested T-cells with OVA expressing cancer cells in a 1:1 ratio. Antigen stimulated OT-1 cells were activated for 3-4 days prior to co-culture experimentation.

**Asymmetric CRISPR double knockout screen**

Library transduced cells resuspended in cRPMI were seeded at a density of 2.5e4 into three 96 well plates to achieve a coverage of ~720X for the R1 screen per E:T ratio. OT-I CD8^+^ T-cells were added to each well at either E:T 2 or 5 and cultured for 48 h before the introduction of 1ug/ml puromycin to remove selective pressure of T-cells. Cells were then allowed to rest for 72 h before being collected for gDNA extraction. The screen was repeated in entirety using the same methodology to have independent experimental biological replicates, with the sole exception of using 6 plates per E:T ratio to increase library coverage.

**Genomic DNA extraction**

To isolate gDNA from CADRE screen cells, cells were washed three times with PBS to remove cellular debris from dead cells before being collected and pooled by condition. Pooled cells were spun down at 400 x *g* for 5 min and reconstituted in 200 μL of PBS before being extracted with Qiagen blood mini gDNA extraction kit according to manufacturer’s protocol. To isolate gDNA for non-screen samples ~2.5e4 cells were taken per sample, and washed with PBS. PBS was aspirated and resuspended in 100 μL Lucigen QuickExtract buffer and incubated for 30 minutes at 65 ℃ before being heat inactivated at 95 ℃ for 5 min.

**CADRE library readout**

Two rounds of PCR were used for sgRNA library readout. PCR #1 used genomic DNA (~2 μg per reaction and 3 reactions per sample) for sufficient coverage of screen, and PCR #2 used 1μL of PCR#1 product with barcoded primers. Samples were amplified with different barcoded primers and pooled for deep sequencing.

The following cycle parameters were used for PCR #1: 98 °C for 1 min, 25 cycles of (98 °C for 1 s, 62 °C for 5 s, 72 °C for 15 s). The following primers were used as well:

Forward: 5’-aatggactatcatatgcttaccgtaacttgaaagtatttcg-3’

Reverse: 5’-ctttagtttgtatgtctgttgctattatgtctactattctttccc-3’

The following cycle parameters were used for PCR #2: 98 °C for 30 s, 28 cycles of (98 °C for 1 s, 62 °C for 5 s, 72 °C for 15 s), and 72 °C 2 min for the final extension. See Table S4 for barcoded primers. PCR reactions were performed using Phusion Flash High Fidelity Master Mix (ThermoFisher). Gel purification of pooled products from a 2% E-gel EX (Life Technologies) were performed using the QiaQuick Gel Extraction kit (Qiagen).

**CADRE library mapping:**

Raw paired FASTQ files were filtered and demultiplexed using Cutadapt ^5^. To demultiplex the barcodes in the forward PCR primers used during readout, the following settings were used cutadapt -g file:fbc.fasta –no-trim, where the fbc.fasta contained the forward barcodes. To pare down the forward read to the first 20 base pair sgRNA spacer sequence, and the reverse read to the second spacer sequence, the following settings were used cutadapt -g GTGGAAAGGACGAAACACCG -G CTCTAAAAC -l 20 -e 0.2 -m 19 –discard-untrimmed. The reverse complement of the second spacer sequence from the reverse read was obtained using fastx_reverse_complement from FASTX-Toolkit, and then combined with the first spacer sequence from the forward read to create 40bp fused spacer-spacer sequences. These 40bp fused sequences were then mapped to the fused sgRNA sequences from the CADRE library (Supplementary Table 2) for dual sgRNA quantification using BWA-ALN ^6^. A BWA index of the sgRNA library was generated using the bwa index command, and SAMTools was used for post-processing ^7^.

**Minimum count threshold**

We determined a minimum count threshold used for downstream analysis (**Figure S1D**). First, we measured frequencies of the SKO sgRNAs in the library, calculated the expected frequencies of the double sgRNAs, and compared them to observed DKO double sgRNAs frequencies. The ratio of observed to expected frequencies fell markedly below a read count of 15. The sgRNAs with counts below this threshold (read count of 15) were then masked from further analysis.

**Identification of gene interaction sgRNA pairs**

We used two methods to identify potential gene interactions. Sample counts were read normalized to 1e6. In order to see if the phenotypic effect of DKO dual gene pair perturbation was different from the constitutive SKO perturbations, we performed the two-sided Wilcoxon rank sum test on DKO sgRNAs abundances compared to gene A and gene B SKO sgRNA abundances for gene pairs. In order to determine enrichment-based gene interactions, we calculated the observed and expected DKO enrichment:

E_observed_ = (D_s_/D_c_) * (N_c_/N_s_)

E_expected_ = (A_s_/A_c_) * (N_c_/N_s_) + (B_s_/B_c_) * (N_c_/N_s_)

D_s_ = median abundance for DKO pair observed in post-selection screen samples

D_c_ = median abundance for DKO pair observed in pre-T cell treatment cell control samples

N_c_ = median abundance for DNTCs observed in pre-T cell treatment cell control samples

N_s_ = median abundance for DNTCs observed in post-selection screen samples

A_s_ = median abundance for SKO gene A observed in post-selection screen samples

A_c_ = median abundance for SKO gene A observed in pre-selection cell control samples

B_s_ = median abundance for SKO gene B observed in post-selection screen samples

B_c_ = median abundance for SKO gene B observed in pre-selection cell control samples

To determine the outlier gene interactions, linear regression was performed on E_expected_ vs E_observed_ using the lm function in R, and the outlier.test function from the “car” package was used to determine Bonferroni p-values for the most extreme observations.

**Luciferase assay for cell survival**

Luciferase readout was done both 24 and 48 h after co-culture in 96-well white polystyrene plates. 150 μg/mL D-luciferin (PerkinElmer) was added using a multichannel pipette to cells, and covered from light and incubated for 10 min. After 10 min, luciferase intensity was measured using a plate reader (PerkinElmer)

**TCGA transcriptome analyses**

For global comparative TCGA gene expression profile analyses, Gene Expression Profiling Interactive Analysis (GEPIA) ^8^ and GEPIA2 ^9^ were used. Log normalized transcripts per million (TPM) values were visualized for normal RNA-seq samples from GTEx and tumor RNA-seq samples from TCGA across 33 different cohorts or selected cohorts using individual gene queries or gene signature queries, with significance thresholds set at |log2FC| cutoff of 0.5 and q-value cutoff at 0.01. Correlation analyses used Spearman’s coefficient. Cell type proportion analysis of GTEx normal and TCGA tumor samples were performed using GEPIA2021 and CIBERSORT for deconvolution.

For additional *KMT2D* correlation analysis, skin cutaneous melanoma (SKCM) TCGA RNA-seq samples were obtained from the Broad GDAC and normalized to TPM. Spearman correlations were calculated using the cor function in R. Genes that were significantly positively or negatively correlated with *KMT2D* were determined using a cutoff to select approximately the top or bottom 10% of the sorted values, and the identified genes were used for Database for Annotation, Visualization and Integrated Discovery (DAVID) ^10,11^ functional gene annotation analysis.

**Survival analyses**

Survival analyses based on the expression status of genes were performed using GEPIA2 and parameters Group Cutoff: Median, Cutoff-High(%): 50; Cutoff-Low(%): 50. Survival maps were also created, using a significance level of 0.05. Survival analyses for how query genes affect the influence of cytotoxic T lymphocyte (CTL) levels on patient outcomes were performed using the Tumor Immune Dysfunction and Exclusion (TIDE) algorithm ^12^.

**Gene mutation profiles in patient cohorts**

*KMT2D, JAK1, JAK2, IFNGR1,* and *TP53* allele frequencies was queried using cBioPortal ^13,14^. All melanoma studies were selected for visualization and analyses, leading to a combined study of 2834 samples from 2781 patients in 15 studies. Alteration frequencies were obtained using a minimum number of total cases threshold of 10, and mutual exclusivity and co-occurrence analyses were obtained for all pairwise combinations. Statistics were determined by cBioPortal.

**Sample size determination.**

Sample size was determined according to the lab's prior work or similar studies in the literature.

**Randomization and blinding statements.**

Regular *in vitro* experiments were not randomized or blinded. High-throughput experiments and analyses were blinded by barcoded metadata.

**Standard statistical analysis.**

Standard statistical analyses were performed using regular statistical methods. GraphPad Prism, Excel and R were used for analyses. Different levels of statistical significance were accessed based on specific p values and type I error cutoffs (0.05, 0.01, 0.001, 0.0001). Details of statistical tests were provided in supplemental information.

**Code availability.**

Codes used for data analysis or generation of the figures related to this study are available from the corresponding author upon reasonable request.

**Supplemental Figures**

**Figure S1. Construction and sequencing of the CADRE CRISPR knockout library in plasmid and transduced cell pools**

(A) Schematic overview of double perturbation CRISPR construct designs. Long oligonucleotides containing paired guide sequences were synthesized and cloned into a lentiviral vector. Subsequently, a gene fragment containing a secondary sgRNA scaffold and mU6 promoter were cloned in between the paired guide sequences to reconstitute two fully functional sgRNA expression systems.

(B) Density plot representing the distribution of scores for cutting efficiency, out-of-frame patterns, and specificity for sgRNAs comprising the CADRE library.

(C) Density plot for the sgRNA pair representation in the CADRE plasmid library.

(D) Minimally required sgRNA pair read count estimation from plasmid library. Observed frequency of double sgRNA was compared to expected frequency calculated from single sgRNAs reads. This analysis revealed that below ~15 read counts, this ratio falls below expected. Guides with less than 15 read counts were masked from further analysis.

(E) Scatterplot comparing guide representation of the CADRE library in the pre-T cell treatment infected cell controls averaged across all replicates and screens to the plasmid control.

**Figure S2. CADRE asymmetric double knockout CRISPR screen revealed dynamic cellular population shifts before and after co-culture selection**

(A) Tukey box plots (IQR boxes with 1.5 × IQR whiskers) overlaid on dot plots of sgRNA pair abundance in samples across screens, E:T ratios, technical replicates, and both pre-T cell treatment (labelled as “cell”) and plasmid controls.

(B) Heatmap showing Pearson correlation of log normalized sgRNA pair abundances across samples.

(C) Empirical cumulative distribution function (CDF) plot of sgRNA pair abundances across samples.

**Figure S3. Additional analysis of CADRE double knockout CRISPR screen on T cell killing**

(A–D) Scatterplots comparing guide representation of the CADRE library in post co-culture samples compared to pre-T cell treatment infected cell controls for (A) screen 1 and E:T ratio 2, (B) screen 1 and E:T ratio 5, (C) screen 2 and E:T ratio 2, (D) screen 2 and E:T ratio 5. *Jak1* and *Jak2* associated sgRNA pairs (either DKO or SKO) are marked in red (JAK), with other non-JAK DKO sgRNA pairs marked in dark blue, other SKO sgRNA pairs marked in light blue, and DNTCs marked in yellow.

(E) Scatter plot of -log10 adjusted P values for each gene pair comparing DKO sgRNAs abundances to constitutive SKO sgRNA abundances by two-sided Wilcoxon rank sum test.

(F) DNTC normalized enrichment of observed DKO phenotype compared to expected DKO phenotype (gene A + gene B enrichment). Linear regression with 0.95 confidence interval shown with significant outliers (Bonferroni adjusted p value < 0.05) with positive residuals marked in blue and negative residuals marked in red.

**Figure S4. Additional analyses of TCGA cancer and matched normal RNA-seq samples**

(A–D) Dot plots showing the gene expression profiles of (A) *KMT2D*, (B) *JAK1*, (C) *TP53*, and (D) *IFNGR1* across all tumor samples and paired normal tissues. Green dots represent normal samples, red dots represent tumor samples. Cohorts with q-value < 0.01 and |log2FC| > 0.5 are labelled in green if expression levels are greater for the normal samples, and in red if levels are greater for the tumor samples.

(E) Kaplan-Meier curves showing the survival of patients from 33 different TCGA cohorts based on the expression status of *KMT2D*. Statistics shown on plot.

(F) Kaplan-Meier curves showing the survival of patients from 33 different TCGA cohorts based on the expression status of *JAK1*. Statistics shown on plot.

(G) Survival map showing the overall survival contributions of *KMT2D*, *JAK1*, *JAK2*, *IFNGR1*, and *TP53* across multiple TCGA cohorts.

(H) Survival map showing disease free survival contributions of *KMT2D*, *JAK1*, *JAK2*, *IFNGR1*, and *TP53* across multiple TCGA cohorts.

**Figure S5. Analyses of TCGA cancer and matched normal RNA-seq samples and mutational profiles**

(A) Scatterplots comparing *KMT2D* expression with (far left) *JAK1* expression in TCGA SKCM dataset; (left) *JAK1* expression in pan-cancer TCGA dataset of 33 types; (right) interferon-gamma-mediated signaling pathway gene signature (gene ontology 0060333) in TCGA SKCM dataset; (far right) interferon-gamma-mediated signaling pathway gene signature in pan-cancer TCGA dataset of 33 types.

(B) Alteration frequencies for *KMT2D* and *JAK1* in 2834 samples from 15 melanoma studies.

(C) Co-mutation analyses for *KMT2D*, *JAK1*, *JAK2*, *IFNGR1*, and *TP53* across melanoma studies. Statistics shown in table.

(D) Kaplan-Meier curves showing the survival of TCGA-SKCM patients based on the expression status of *KMT2D*. Statistics shown on plot.

(E) Kaplan-Meier curves showing the survival of TCGA-SKCM patients based on the expression status of *JAK1*. Statistics shown on plot.

**List of Supplemental Tables (provided in a compound excel file)**

S1. CADRE dual sgRNA library by gene pair.

S2. Concatenated sgRNA sequences for mapping CADRE.

S3. SgRNA quality scoring by CRISPOR.

S4. Second PCR NGC barcoded readout primers.

S5. CADRE abundance counts for plasmid, cell control, and screens.

S6. Wilcoxon rank sum test significance for DKO dual sgRNA construct abundance compared to SKO gene A abundance or SKO gene B abundance.

S7. Observed DKO dual sgRNA construct enrichment compared to expected enrichment determined by SKO gene A enrichment + SKO gene B enrichment.

S8. Studentized residual vs significance from gene pair outlier test on linear fit of observed vs expected DKO enrichment.

S9. Spearman correlation values for comparisons to *KMT2D* with gene expression values from TCGA SKCM melanoma samples.

S10. DAVID analysis for genes negatively correlated with *KMT2D* in TCGA SKCM samples.

S11. DAVID analysis for genes positively correlated with *KMT2D* in TCGA SKCM samples.

**Supplemental References:**

1. Kandoth, C. *et al.* Mutational landscape and significance across 12 major cancer types. *Nature* **502**, 333–339 (2013).

2. Haeussler, M. *et al.* Evaluation of off-target and on-target scoring algorithms and integration into the guide RNA selection tool CRISPOR. *Genome Biol.* **17**, 148 (2016).

3. Ye, L. *et al.* In vivo CRISPR screening in CD8 T cells with AAV– Sleeping Beauty hybrid vectors identifies membrane targets for improving immunotherapy for glioblastoma. *Nat. Biotechnol.* **37**, 1302–1313 (2019).

4. Dong, M. B. *et al.* Systematic Immunotherapy Target Discovery Using Genome-Scale In Vivo CRISPR Screens in CD8 T Cells. *Cell* **178**, 1189-1204.e23 (2019).

5. Martin, M. Cutadapt removes adapter sequences from high-throughput sequencing reads. *EMBnet.journal* **17**, 10–12 (2011).

6. Li, H. & Durbin, R. Fast and accurate short read alignment with Burrows–Wheeler transform. *Bioinformatics* **25**, 1754–1760 (2009).

7. Li, H. *et al.* The Sequence Alignment/Map format and SAMtools. *Bioinformatics* **25**, 2078–2079 (2009).

8. Tang, Z. *et al.* GEPIA: a web server for cancer and normal gene expression profiling and interactive analyses. *Nucleic Acids Res.* **45**, W98–W102 (2017).

9. Tang, Z., Kang, B., Li, C., Chen, T. & Zhang, Z. GEPIA2: an enhanced web server for large-scale expression profiling and interactive analysis. *Nucleic Acids Res.* **47**, W556–W560 (2019).

10. Huang, D. W., Sherman, B. T. & Lempicki, R. A. Systematic and integrative analysis of large gene lists using DAVID bioinformatics resources. *Nat. Protoc.* **4**, 44–57 (2009).

11. Huang, D. W., Sherman, B. T. & Lempicki, R. A. Bioinformatics enrichment tools: paths toward the comprehensive functional analysis of large gene lists. *Nucleic Acids Res.* **37**, 1–13 (2009).

12. Jiang, P. *et al.* Signatures of T cell dysfunction and exclusion predict cancer immunotherapy response. *Nat. Med.* **24**, 1550–1558 (2018).

13. Cerami, E. *et al.* The cBio Cancer Genomics Portal: An Open Platform for Exploring Multidimensional Cancer Genomics Data. *Cancer Discov.* **2**, 401–404 (2012).

14. Gao, J. *et al.* Integrative Analysis of Complex Cancer Genomics and Clinical Profiles Using the cBioPortal. *Sci. Signal.* **6**, pl1–pl1 (2013).
